# Biocompatibility and Biological Efficiency of Inorganic Calcium Filled Bacterial Cellulose Based Hydrogel Scaffolds for Bone Bioengineering

**DOI:** 10.3390/ijms19123980

**Published:** 2018-12-11

**Authors:** Probal Basu, Nabanita Saha, Radostina Alexandrova, Boyka Andonova-Lilova, Milena Georgieva, George Miloshev, Petr Saha

**Affiliations:** 1 Centre of Polymer Systems, University Institute, Tomas Bata University in Zlin, Trida Tomase Bati 5678, 760 01 Zlín, Czech Republic; probal@utb.cz (P.B.); saha@utb.cz (P.S.); 2 Institute of Experimental Morphology, Pathology and Anthropology with Museum, Bulgarian Academy of Sciences, 1113 Sofia, Bulgaria; rialexandrova@hotmail.com (R.A.); boika_andonova@hotmail.com (B.A.-L.); 3 Laboratory of Molecular Genetics, Institute of Molecular Biology “Acad. R. Tsanev”, Bulgarian Academy of Sciences, 1113 Sofia, Bulgaria; milenageorgy@gmail.com (M.G.); miloshev@bio21.bas.bg (G.M.)

**Keywords:** bacterial cellulose, *in vitro* bio-mineralization, bone tissue engineering, biocompatibility, apoptosis, DNA damage

## Abstract

The principal focus of this work is the in-depth analysis of the biological efficiency of inorganic calcium-filled bacterial cellulose (BC) based hydrogel scaffolds for their future use in bone tissue engineering/bioengineering. Inorganic calcium was filled in the form of calcium phosphate (β-tri calcium phosphate (β-TCP) and hydroxyapatite (HA)) and calcium carbonate (CaCO_3_). The additional calcium, CaCO_3_ was incorporated following *in vitro* bio-mineralization. Cell viability study was performed with the extracts of BC based hydrogel scaffolds: BC-PVP, BC-CMC; BC-PVP-β-TCP/HA, BC-CMC-β-TCP/HA and BC-PVP-β-TCP/HA-CaCO_3_, BC-CMC-β-TCP/HA-CaCO_3_; respectively. The biocompatibility study was performed with two different cell lines, i.e., human fibroblasts, Lep-3 and mouse bone explant cells. Each hydrogel scaffold has facilitated notable growth and proliferation in presence of these two cell types. Nevertheless, the percentage of DNA strand breaks was higher when cells were treated with BC-CMC based scaffolds i.e., BC-CMC-β-TCP/HA and BC-CMC-β-TCP/HA-CaCO_3_. On the other hand, the apoptosis of human fibroblasts, Lep-3 was insignificant in BC-PVP-β-TCP/HA. The scanning electron microscopy confirmed the efficient adhesion and growth of Lep-3 cells throughout the surface of BC-PVP and BC-PVP-β-TCP/HA. Hence, among all inorganic calcium filled hydrogel scaffolds, ‘BC-PVP-β-TCP/HA’ was recommended as an efficient tissue engineering scaffold which could facilitate the musculoskeletal (i.e., bone tissue) engineering/bioengineering.

## 1. Introduction

Bone is an important part of the animal musculoskeletal system. The structural framework of an animal is preserved by the bones through modelling and remodeling events [1,2]. Extensive research indicated that bone related disorders like osteoporosis affect 75 million individuals throughout Europe, USA and Japan. In addition, many studies also showed that osteoporosis causes more than 8.9 million fractures worldwide annually; with a condition where an osteoporotic fracture occurs in every 3s [3]. The possible treatment methods for this comprise the use of either auto/allografts or ceramic coated/inert metallic implants, which in many cases are far too expensive for application [4]. In this context, the hydrogel based bioactive scaffold can become a notable approach in bone tissue engineering/bone bioengineering; due to its osteo-conduction and osteo-induction properties, notable mechanical property and further its cost-effective production attributes [5].

The hydrogel is a three dimensional polymeric network structure which can retain significant amount of water [6,7,8]. The hydrogel based bioactive scaffolds have the necessary attributes to become an efficient extra cellular matrix (ECM) that has the potential to execute the primary functions of the tissue engineering scaffolds like cell adhesion, stimulation for cell proliferation and others [9,10]. Different polymers, polymer-composite scaffolds are often utilized in the design of an efficient scaffold material. Additionally, a variety of synthetic polymers like poly(lactic-co-glycolic) acid (PLGA), poly(glycolic acid) (PGA) poly(caprolactone) (PCL) and natural polymers like collagen, hyaluronic acid have also been used in the fabrication of tissue engineering scaffold [11,12,13,14,15,16].

Research showed that bacterial cellulose (BC) based hydrogel scaffolds could also become a potential biomaterial for tissue regeneration application [17]. BC is a biocompatible biopolymer [18] and has high crystallinity, ultra-fine network structure and high water absorption capability [19,20]. These significant structural and functional properties of BC increase its importance in musculosketal/tissue engineering/bone bioengineering applications.

The inorganic phase of the bone tissue is composed majorly of calcium mineral [21]. However, recent research data reported that the extracellular calcium had a significant role in cellular growth and development [22]. Bone cells are comprised of different calcium ion channels and extracellular calcium receptors that receive the signals from the extracellular Ca^2+^ [23,24], which in turn generates specific genetic responses related to cell growth and proliferation [25]. Studies indicated that the biocompatibility and the mechanical properties of the tissue engineering scaffolds can be modified and improved by addition of calcium phosphate [26,27]. Bioactive calcium phosphate fillers like β-tri-calcium phosphate (β-TCP), octa-calcium phosphate (OCP) and hydroxyapatite (HA) improve the osteo-conduction and osteo-induction properties of the biomaterial [17,28]. On the other hand, inorganic calcium can also be incorporated in the tissue engineering scaffold through organic-inorganic hybridization. A variety of methods of the organic-inorganic hybridization (i.e., solvent casting/particle leaching, scaffold coating, etc.) have been developed for the inclusion of bioactive CaCO_3_ within the polymer matrix in order to obtain improvement in the structural and functional properties of the scaffold [29].

Polyvinylpyrrolidone (PVP) is a synthetic polymer which has significant biocompatibility. Several studies indicated that the application of PVP is not so widespread due to its poor mechanical properties and low swelling capacity [30]. However, the properties of PVP can be improved when it is blended with polysaccharides. On the other hand, carboxymethyl cellulose (CMC) is the cellulose derivative which has also significant utilization in cosmetology and as a water retention agent. Additionally, CMC has notable biocompatibility [30,31]. The blending of the above mentioned polymers, PVP-CMC hydrogel scaffold; has been previously successfully prepared in our laboratory. The biological efficiency of PVP-CMC scaffold has also been found suggestive [30,31,32]. BC is a natural polymer which also has significant biocompatibility and notable mechanical properties. Recent data indicated that a variety of composite materials prepared with BC such as BC/Chitosan, BC/collagen were used in biomedical applications; especially in tissue engineering/bone bioengineering [33,34]. The hydrogel scaffolds prepared from the natural polymers like BC and synthetic polymers PVP and CMC have also been reported in our previous work [19]. Albeit all the above-mentioned hydrogel scaffolds have the necessary material characteristics (like mechanical, rheological properties) for tissue engineering application; however, in order to develop a more specific bone tissue engineering scaffold, inorganic calcium (in the form of calcium phosphate or/and CaCO_3_) filled BC based hydrogel scaffolds were developed. In the present work, the focus is given on the *in vitro* characterization of the novel inorganic calcium filled BC based hydrogel scaffolds through evaluation of their biocompatibility and biological efficiency in a series of cell-based assays.

## 2. Results

The novel inorganic calcium filled scaffolds “BC-PVP-β-TCP/HA”, “BC-CMC-β-TCP/HA”, “BC-PVP-β-TCP/HA-CaCO_3_”, and “BC-CMC-β-TCP/HA-CaCO_3_” were prepared and their biocompatibility and biological efficiency (in a series of cell based assays) were evaluated in order to recommend for its application for bone tissue engineering/bioengineering, where, the “BC-PVP” and “BC-CMC” scaffolds were considered as a control set of scaffolds. To analyze the biological efficiency of these scaffolds, the following investigations were performed and discussed below.

### 2.1. Cell Viability and Biocompatibility Study

**Figure 1:**
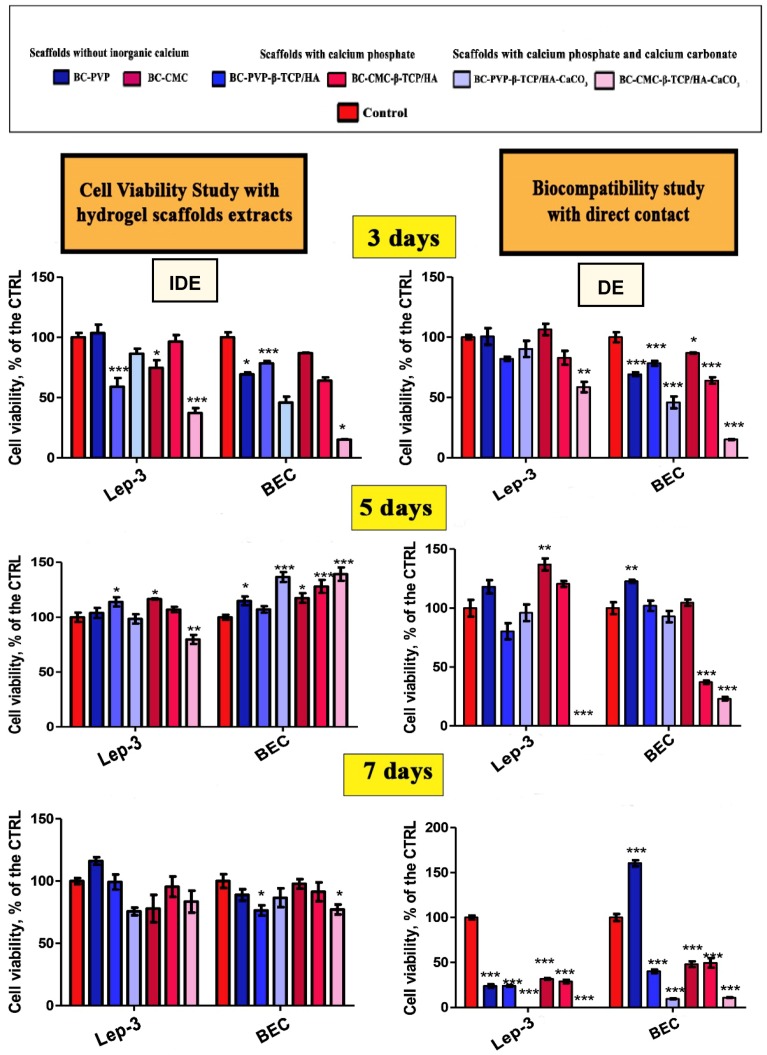
Human fibroblast, Lep-3 and mouse bone explant cells (BEC) cellular viability (proliferation profiles) after incubation with six different BC based hydrogel scaffolds (scaffolds without inorganic calcium, scaffolds with calcium phosphate, scaffolds with calcium phosphate and CaCO_3_); established by indirect (IDE) and direct experiments (DE). * *p* < 0.05; ** *p*< 0.005; *** *p* < 0.0001 as compared to the control.

Cell viability studies have been performed through MTT(3-(4,5-Dimethylthiazol-2-yl)-2,5-diphenyltetrazolium bromide) test in indirect (IDE) and direct experiments (DE) using human embryonic fibroblasts (Lep-3) and mouse bone explant cells (BEC), and are summarized in Figure 1.

It can be seen from Figure 1 that the detected cytotoxicity of the calcium filled scaffolds was low in the studied cell cultures, when determined in IDEs and incubated in 3, 5 and 7 days’ extract culture medium. In contrast, it is clearly visible that the existence of the viable Lep-3 and BEC cells has been specially affected by the presence of BC-CMC-β-TCP/HA-CaCO_3_ and decreased in number after 5 days. However, both the cells were affected more or less in the presence of all the scaffolds after 7 days except “BC-PVP-β-TCP/HA” in DE and in presence of BEC. In general, “BC-CMC-β-TCP/HA-CaCO_3_” exhibited the highest cytotoxic activity in DEs for both human and mouse cells (i.e., Lep-3 and BEC).

### 2.2. Genotoxic Potential of the Studied BC Based Inorganic Calcium Filled Hydrogel Scaffolds

**Figure 2:**
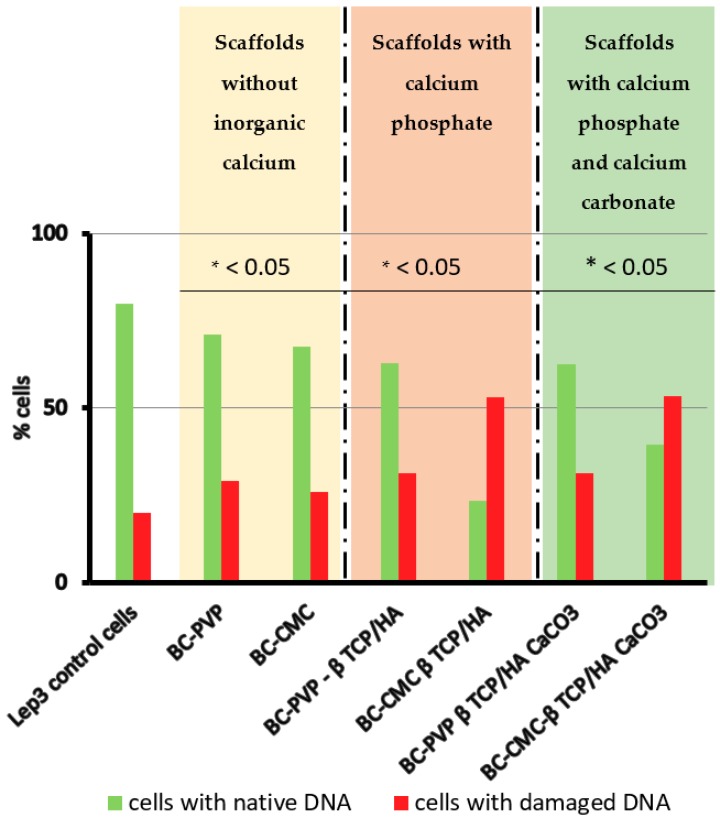
Genotoxicity testing of the studied biocompatible scaffolds was done by the method of Comet Assay in Lep-3 cells in the presence of extracts of all six BC based hydrogel scaffolds. Data quantitation proceeded with estimation of the percentage of comets by assuming the total number of objects per probe (*n* = 1000) as 100% and the percentage of comets, i.e., cells with damaged DNA was estimated and represented as a graph. Student *t* test was performed and the estimated *p* values for all probes in comparison to the control were statistically significant *p* < 0.05.

The genotoxic potential of the studied BC based inorganic calcium filled hydrogel scaffolds was determined by the method of Comet Assay in alkaline conditions in order to detect all kinds of DNA breaks; in which the tested scaffolds can potentially induce genotoxic effect (i.e., DNA breaks) in the cells. Lep-3 cells were used as the model cellular system in this study. The Comet Assay data quantitation included calculation of the percentage of comets, i.e., cells with damaged DNA in all tested probes and results are displayed on Figure 2. It can be seen that, the higher percentage of comets (cells with damaged DNA) was observed in Lep-3 cells grown in 3 days’ extract culture medium from BC-CMC-β-TCP/HA and BC-CMC-β-TCP/HA-CaCO_3_; where more than 60% of all observed cells per probe were with damaged DNA. On the other hand, more than 60% of cells incubated with BC-PVP-β-TCP/HA and BC-PVP-β-TCP/HA-CaCO_3_ were found with native, i.e., untouched/unaffected DNA, in comparison to the control and cells with BC-CMC-β-TCP/HA and BC-CMC-β-TCP/HA-CaCO_3_ scaffolds.

### 2.3. Study on Apoptosis/Necrosis

**Figure 3:**
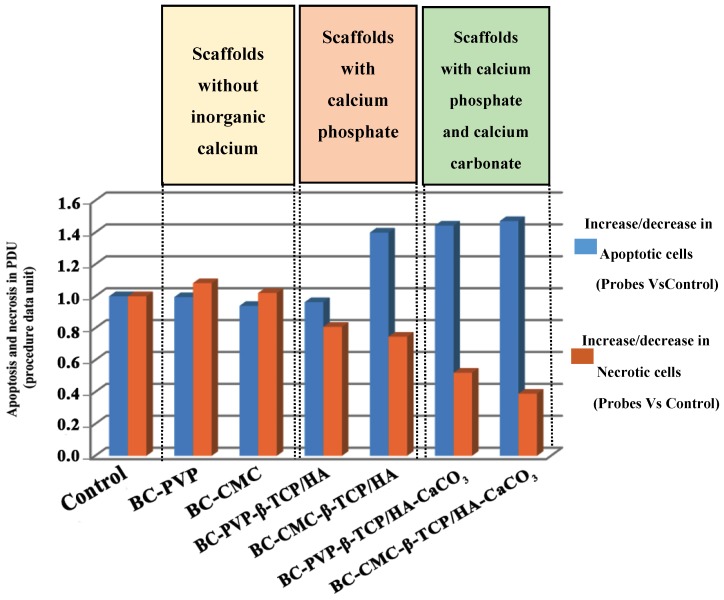
Discrimination between apoptotic and necrotic types of cell death induced by the tested scaffolds in Lep-3 cells. FACS analysis with Annexin V-FITC kit for apoptosis/necrosis detection was applied on Lep-3 cells cultivated in presence of six studied scaffolds. Data quantitation included estimation of the percentage of cells undergoing apoptosis and necrosis and the ratio between these percentages in comparison to the control cells is given in PDU (procedure data units).

In order to elucidate the mechanisms of the biological action of the tested scaffolds on the studied cellular model systems and to understand the reasons for the detected genotoxic potential of those with calcium phosphate and carbonate functionalization, i.e., BC-CMC-β-TCP/HA and BC-CMC-β-TCP/HA-CaCO_3_ (already detected with the Comet Assay Figure 2), FACS analysis (fluorescence activated cell sorting) with Annexin V-FITC kit performed for evaluation of the percentage of cells undergoing apoptosis and necrosis when layered onto all studied scaffolds. The percentage of apoptotic and necrotic cells together with the percentage of live cells was estimated by software data quantitation. Further on the ratio among cells in apoptosis and necrosis and alive cells was done and is displayed in procedure data values (PDU) on Figure 3. The last represents the ratio between apoptotic and necrotic cells in the probes versus these percentages in the controls (Lep-3 control cells). The blue bars on Figure 3 represent the ratio between the percentages of apoptotic cells in the probes versus the control, while the red bars represent the ratio between the percentages of necrotic cells in the tested probes versus the necrotic cells in the control. As it is easily seen on Figure 3 an increased number (around 40%) of apoptotic cells was detected in the probes of human Lep-3 cells grown in 3 days’ extract culture medium in presence of BC-CMC-β-TCP/HA, BC-PVP-β-TCP/HA-CaCO_3_ and BC-CMC-β-TCP/HA-CaCO_3_. Interestingly, the trend with the observed increase of apoptosis between BC-PVP and BC-PVP-β-TCP/HA was similar but when compared to BC-CMC-β-TCP/HA, BC-PVP-β-TCP/HA-CaCO_3_ and BC-CMC-β-TCP/HA-CaCO_3_ it was not so-well pronounced as previously expected.

Noteworthy, the results from the FACS analysis for discrimination between apoptosis and necrosis are in correspondence with the results from the alkaline Comet Assay. The detected genotoxic potential of BC-CMC-β-TCP/HA and BC-CMC-β-TCP/HA-CaCO_3_ is also reflected in the experiments for studying of the mechanisms for the induction of different types of cell death. In the cases when cells were cultivated in the presence of these types of scaffolds (i.e., BC-CMC-β-TCP/HA and BC-CMC-β-TCP/HA-CaCO_3_), the higher levels of apoptosis were seen. It could be a result from the possible genotoxic effect of these substances/scaffolds.

### 2.4. SEM Analysis

**Figure 4:**
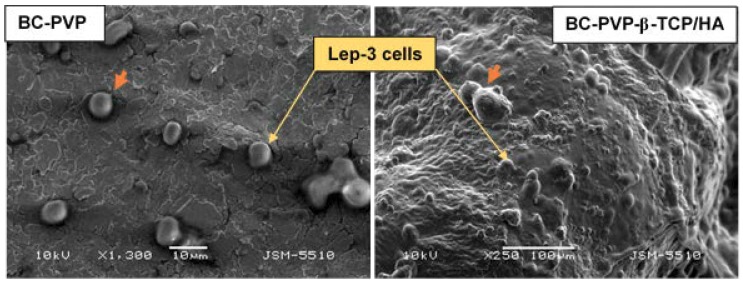
SEM images of Lep-3 cells grown on BC based hydrogel (BC-PVP and BC-PVP-β-TCP/HA). The orange colored arrow indicates developing projections.

The SEM analysis of hydrogel scaffolds-Lep-3 cell interaction is depicted in Figure 4. The analysis was performed with BC-PVP and BC-PVP-β-TCP/HA, as these materials showed better cyto-compatibility in direct (DE) and indirect experiments (IDE). The presence of Lep-3 cells including groups of 2–3 cells was observed on the surface of both materials (Figure 4). The adhered Lep-3 cells on the surface of BC-PVP-β-TCP/HA can be seen notably.

## 3. Discussion

Generally, the significant properties of scaffold materials involve efficient architecture, cyto-compatibility and notable mechanical property. Recently many different scaffold materials have been developed with a variety of synthetic and natural polymers [11]. However, the application of natural polymers like bacterial cellulose (BC) for tissue regeneration is also very promising [35]. In this study, BC was used with different biocompatible polymers like PVP, CMC, etc. Calcium phosphate (in the form of β-TCP/HA) was added within the polymer matrix. Moreover, the calcium phosphate filled scaffolds were further *in vitro* biomineralized to develop calcium phosphate and calcium carbonate filled biomineralized hydrogel scaffold. The applied cell based assays for studying of their biological properties demonstrated the promising cyto-compatibility.

Calcium phosphate like β-TCP and HA can facilitate osteo-conduction, which ultimately results in high cell viability [36]. In the cell viability study with sample extract in IDE, the BEC viability was significant for BC-PVP-β-TCP/HA-CaCO_3_ after 5 days of incubation. Additionally, there was a trend of increase also seen in BEC viability with BC-CMC and calcium phosphate and calcium carbonate filled hydrogel scaffolds after 5 day of incubation. Moreover, until the 7 day of incubation, cell viability for Lep-3 and BEC was still significant for all the BC based hydrogels. Other authors studies indicated that CaCO_3_ facilitates cell viability for fibroblasts [37]. In this context, it is relevant to mention that; the Lep-3 cells are embryonic fibroblast cells [38] and not at all are specified to elicit prompt response in the presence of notable calcium content in the surrounding environment. Thus, through acclimatization with the significant inorganic calcium environment, the cells exhibit high viability with the gradually increasing incubation period from 72 to 168 h. In regard to the biocompatibility study in the direct contact (DE) with the scaffolds, the BEC and Lep-3 cells initially acclimatized with the chemical and physical environment that has been provided by the scaffold sections and thus showed cell growth and viability after 3 days of incubation. Then gradually the higher cell viability started to be visible after 5 days of incubation. Interestingly, after 7 days of incubation, the Lep-3 and BEC cell viability decreased in comparison to cell viability after 3 days and 5 days of incubation. In any case, the BEC viability was found higher for BC-PVP after 7 days of incubation.

Porosity is an important factor for bone cell growth and proliferation [39]. Thus, the significant porous structures of BC-PVP scaffold section might provide the necessary environment for BEC growth efficiently after 7 days of incubation. On the other hand, research indicated that high levels of Ca^2+^ can negatively influence DNA repair ability to the cells [40] and thereby can cause less cell viability. Interestingly, after 7 days of incubation, the BEC and Lep-3 cell viability was significantly fewer when incubation performed with *in vitro* biomineralized samples. Lep-3 cells were unable to get any proliferation stimulation in contact (DE) with the *in vitro* biomineralized samples (BC-PVP-β-TCP/HA-CaCO_3_, BC-CMC-β-TCP/HA-CaCO_3_) after 7 days of incubation. This indicates that *in vitro* biomineralized hydrogels can only provide an initial stimulatory signals to Lep-3 cells. As Lep-3 cells are sensitive human embryonic cells (but not stem cells), the initial high concentration of calcium in the cell culture environment causes them to significantly proliferate up to 3 days of incubation in the presence of all six hydrogel scaffolds, while no proliferation was observed after 5 days of incubation during direct experiments (DE). However, the Lep-3 cell viability after 5 days of incubation with BC-PVP-β-TCP/HA-CaCO_3_ very possibly resulted from the combined positive effect provided by the polymers (BC and PVP) and the inorganic calcium content. Finally, the high calcium content most probably inhibits the subsequent proliferation of both human and mouse cells after 7 days of incubation.

The results from the method of comet assay signified the occurrence of DNA damage in the cells when Lep-3 cells were used in the study to evaluate the genotoxic effect of hydrogel scaffolds. Albeit, BC and CMC have been proved to be biocompatible polymers, together with calcium phosphate in BC-CMC-β-TCP/HA hydrogel scaffold, they might develop a cellular burden by possible induction of considerable number of DNA breaks. Earlier reports showed that several polymers and ceramics can indeed cause oxidative stress [41,42] in the cells, which ultimately resulted in DNA strand breaks [43]. The possible interaction of BC, CMC and calcium phosphate (for BC-CMC-β-TCP/HA hydrogel scaffold) might develop oxidative stress to the cells which could finally result in high percentage of comets (cells with damaged DNA). Moreover, research indicated that the elevation of the calcium ion (Ca^2+^) concentration can also influence DNA repair ability of the cells [40]. This phenomenon is evident of Figure 1, where the percentage of live cells was found less for the BC-CMC-β-TCP/HA-CaCO_3_ hydrogel scaffold. The degree of DNA damage might be greater than the DNA repair ability for BC-CMC-β-TCP/HA-CaCO_3_. On the other hand, the notable percentage of live cells for BC-PVP-β-TCP/HA and BC-PVP-β-TCP/HA-CaCO_3_ indicated the introduction of less DNA damage into the fibroblast cells (Lep-3).

Regarding apoptosis/necrosis study, the necrosis level was found gradually decreasing compared to apoptosis (Necrosis: BC-PVP > BC-CMC > BC-PVP-β-TCP/HA > BC-CMC-β-TCP/HA > BC-PVP-β-TCP/HA-CaCO_3_ > BC-CMC-β-TCP/HA-CaCO_3_). Other researchers demonstrated that, an elevated extracellular Ca^2+^ can cause the production of reactive oxygen species (ROS) in cellular mitochondria, which thereby induces the destruction of the mitochondrial membrane that finally initiates the apoptosis through the release of cytochrome C into the cytoplasm [44]. Possibly, the *in vitro* biomineralized CaCO_3_ filled samples (BC-PVP-β-TCP/HA-CaCO_3_, BC-CMC-β-TCP/HA-CaCO_3_), creates high Ca^2+^ environment around the Lep-3 cell population which might also increase the intracellular Ca^2+^ levels which could have resulted in apoptosis. Earlier research indicated that caspase-8; a cysteine aspartate protease involved in apoptosis, has a specific role in suppression of necrosis and facilitation of apoptosis [45]. Our studies show that, the apoptosis level is also seen increasing following incubation time with the calcium phosphate filled scaffolds and with the *in vitro* biomineralized CaCO_3_ filled hydrogel scaffolds. The possible formation of caspase-8 in some population of the cells might be responsible for the significant shift from necrosis to apoptosis in all biomineralized CaCO_3_ filled hydrogel scaffolds. Interestingly, the elevated level of apoptosis compared to necrosis can also be seen in BC-CMC-β-TCP/HA compared to BC-PVP-β-TCP/HA hydrogel scaffold. This might be associated with the combinatorial effect of polymers (BC, CMC) and calcium phosphate (β-TCP/HA). In addition, the cell viability, rather than apoptotic/necrotic cell death is also found higher in BC-PVP-β-TCP/HA, compared to BC-CMC-β-TCP/HA and BC-CMC-β-TCP/HA-CaCO_3_. Furthermore, the apoptotic/necrotic cell death is also found 10 folds less for BC-CMC than BC-PVP.

Scientific data revealed that different materials contains various surface characteristics like the surface topography, wettability, roughness, softness which notably influence a variety of cell behavior like cellular adhesion, growth, differentiation [46,47]. BC-PVP and BC-PVP-β-TCP/HA hydrogel scaffolds contain the necessary attributes (mechanical and microstructural properties) [48] which facilitate efficient Lep-3 fibroblast cell adhesion on the surface of the hydrogel scaffolds. Additionally, the homogenous cell adhesion also signifies the efficient adsorption of cell adhesion proteins to the surface of the hydrogel scaffolds. This focal adhesion of cells on the surface of these hydrogel scaffolds will further facilitate the cell proliferation and growth events which ultimately elaborate the event of tissue regeneration [45].

## 4. Materials and Methods

### 4.1. Materials

Polyvinylpyrrolidone K30 (PVP K30; molecular weight: 40,000), Polyethylene glycol 3000 (PEG; average molecular weight: 2700–3300), Agar, β-tri calcium phosphate (β-TCP; molecular weight: 310.18 g/mol) were supplied by Fluka, Switzerland; Sodium carboxymethyl cellulose (CMC) was purchased from Sinopharm Chemical Reagent Co Ltd. (SCRC), China; anhydrous Calcium chloride (CaCl_2_; molecular weight 110.99 g/mol, 97.0%) was obtained from Penta, Czech Republic; and Sodium carbonate-decahydrate (Na_2_CO_3_; molecular weight 286.14 g/mol), Hydroxyapatite (HA; molecular weight: 502.31 g/mol) were obtained from Sigma Aldrich.

Dulbecco’s modified Eagle’s medium (DMEM) and Fetal bovine serum (FBS) were obtained from Gibco-Invitrogen (Loughborough, UK). Thiazolyl blue tetrazolium bromide (MTT). Dimethyl sulfoxide (DMSO) and Trypsin were obtained from AppliChem (Darmstadt, Germany). Agar is supplied by Fluka, Switzerland. The antibiotics (Penicillin and Streptomycin) for cell cultures were from Lonza (Verviers, Belgium). Ethylenediaminetetraacetic acid (EDTA), Glutaraldehyde, Ammonium hydroxide, Formaldehyde and all other chemicals of the highest purity commercially available were purchased from local agents and distributors. All sterile plastic ware and syringe filters were from Orange Scientific (Braine-l′ Alleud, Belgium). Agarose (low-gelling) was purchased from Sigma-Aldrich. ANNEXINV—GFP-Certified Apoptosis/Necrosis detection kit was used from Enzo Life Sciences (Long Island, NY, USA).

### 4.2. Synthesis and Preparation of Homogenous Suspension of BC

BC (holding 99% H_2_O) was synthesized in presence of basal synthetic Hestrin-Schramm (HS) nutritive medium (pH 7.0) using *Gluconacetobacter xylinus CCM 3611^T^* (syn. *Acetobacter xylinum*) at 30 °C for 15 days. 100 mL bacteriological culture bottles were inoculated with 5 mL of H.S. medium containing 96 × 10^8^ cells/mL bacteria (bacteria counted at 550 nm wavelength with Grant-Bio McFarland Densitometer DEN-1B, Grant Instruments Ltd., UK). The freshly prepared BC pellicle is treated with 0.5 N NaOH solution and then heated at 80 °C for 1 h to remove the possible contaminations from the BC pellicle. Thereafter, a homogenous suspension of BC (particle size: 351.03 nm) from the obtained BC mat was prepared by grinding the BC mat in distilled water.

### 4.3. Preparation of Inorganic Calcium Phosphate Filled BC Based Hydrogel Scaffold

BC based hydrogel scaffolds were first developed by applying the BC (holding 99% water) with CMC and PVP (Table 1) [49]. Polyethylene glycol (PEG) was also used in all the solutions to reduce the risk of tissue damage and other significant cytotoxic effects [50]. Agar used as gelling agent and glycerin used as humectant [49].

**Figure 5:**
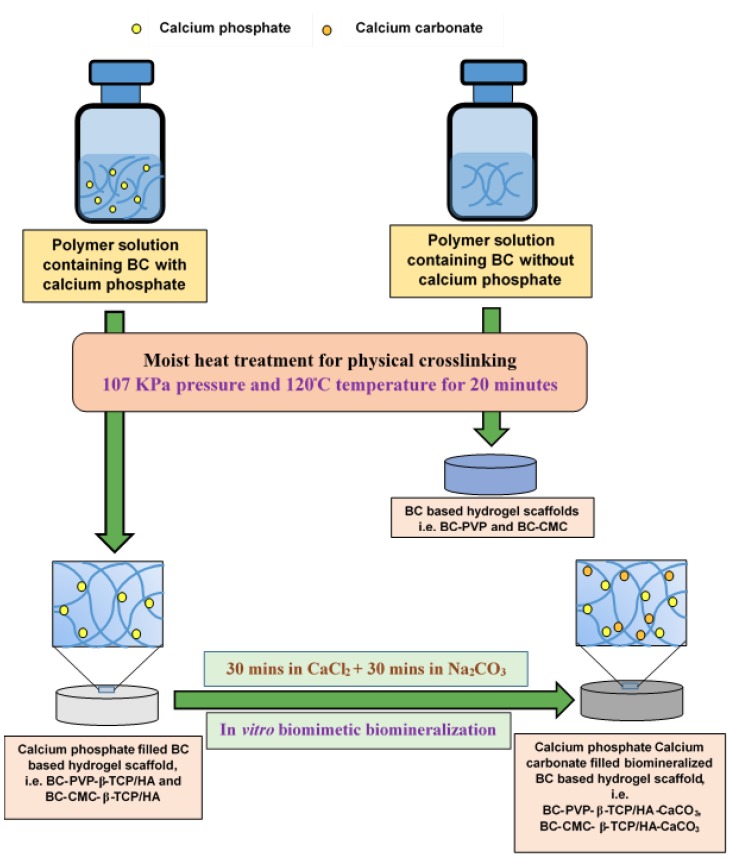
Schematic diagram: preparation of inorganic calcium filled BC based hydrogel scaffolds.

Additionally, β-TCP and HA were applied in the ratio of 20:80 [49,51] in the hydrogel scaffolds to produce the inorganic calcium phosphate filled BC based hydrogel scaffolds. The prepared scaffolds were termed as, “BC-PVP-β-TCP/HA” and “BC-CMC-β-TCP/HA” (Figure 5), where, BC-PVP and BC-CMC hydrogel scaffolds were used as base scaffolds/control set [19].

The BC based hydrogel scaffolds were prepared following the solvent casting method, applying moist heat and pressure. 100 mL polymer solutions were prepared in 250 mL sealed glass bottles under 15 lbs (107 KPa) pressure and 120 °C temperature for 20 min [6]. Two sets of polymer solutions were prepared; where one set was without inorganic calcium phosphate, and another set was with inorganic calcium phosphate (β-TCP/HA). 25 mL polymer solution from each sealed glass bottles was poured into 75 mm diameter petri-dishes and allowed to cool at room temperature (22–25 °C). Finally, smooth, round shaped, off white color BC based scaffold without inorganic calcium phosphate (“BC-PVP” and “BC-CMC”) were achieved; diameter: 75 mm; thickness: 6.0–6.2 mm in wet state. The inorganic calcium phosphate filled scaffolds are termed as “BC-PVP-β-TCP/HA” and “BC-CMC-β-TCP/HA”; diameter: 75 mm; thickness: 5.5–6 mm of hydrogel scaffolds were attained.

### 4.4. Preparation of Biomineralized Inorganic Calcium Phosphate and Calcium Carbonate (CaCO_3_) Filled BC Based Hydrogel Scaffold

The calcium phosphate filled BC based hydrogel scaffolds were further bio-mineralized for incorporation of additional CaCO_3_ for the development of strong bioactive polymeric scaffold material. For achieving that, the *in vitro* bio-mineralization process was performed following the simple liquid diffusion technique [52]. Two different ionic solutions (i.e., Na_2_CO_3_ and CaCl_2_) were used at fixed concentration ratios (5.25/100 mL and 7.35/100 mL) to prepare inorganic calcium phosphate and CaCO_3_ filled BC based hydrogel scaffolds. Research demonstrated that the deposition and accumulation of CaCO_3_ within the hydrogel scaffold is notable between 60–90 min [29]. Thus, in this study, the bio-mineralization of hydrogel matrix was carried out for 60 min by keeping the test samples in each ionic solution. The calcium phosphate filled BC based hydrogel scaffolds were first immersed in 100 mL solution of CaCl_2_·H_2_O for 30 min and then transferred into 100 mL of Na_2_CO_3_ solution and kept them for 30 min. In this procedure, calcium phosphate and CaCO_3_ filled BC based *in vitro* bio-mineralized hydrogel scaffold were achieved having diameter: 75 mm; thickness: 4.5–4.9 mm (in wet state), and as a result, they are finally termed as “BC-PVP-β-TCP/HA-CaCO_3_” and “BC-CMC-β-TCP/HA-CaCO_3_” respectively (Figure 5).

### 4.5. Cell cultures

Human diploid fibroblast; Lep-3 cells from lung of 3-month old human embryo and cell culture established from bone explants of 2–3 months old ICR mice (Bone explant cells, BEC) as it was earlier described [39] were used as model systems in our study. Lep-3 and BEC cells were obtained from the Cell culture collection of the Institute of Experimental Morphology, Pathology and Anthropology with Museum—Bulgarian Academy of Sciences (IEMPAM-BAS).

Both the cultures were grown in DMEM medium supplemented with 10% fetal bovine serum, 100 U/mL penicillin and 100 μg/mL streptomycin. The cultures were kept in a humidified incubator (Thermo Scientific, HEPA Class 100, Waltham, MA, USA) at 37 °C under 5% CO_2_ in air. For routine passages, the cells were detached using a mixture of 0.05% trypsin and 0.02% EDTA. The cell cultures were passaged 2–3 times per week (1:2 to 1:3 split). The experiments were performed during the exponential phase of cell growth. During investigations performed the BEC cells were on their 44–50th passages.

### 4.6. Evaluation of Cell Viability and Proliferation

#### 4.6.1. Sample Preparation

Circular sections (size: 6 mm in diameter, 2–3 mm thickness) of the six freeze dried samples (BC-PVP, BC-CMC, BC-PVP-β-TCP/HA, BC-CMC-β-TCP/HA, BC-PVP-β-TCP/HA-CaCO_3_, BC-CMC-β-TCP/HA-CaCO_3_) were taken for this study. Sections were placed in the 48-well cell culture plate, treated with 50 µL of 96% ethanol for 40 min after which the ethanol was removed and dried at 30–32 °C to complete dryness. Then, the materials were sterilized under the exposure of UV radiation for 80–90 min.

Indirect and direct experiments were performed for evaluating the influence of the materials on cell viability and proliferation

#### 4.6.2. Indirect Experiments (IDE)

Each material was placed in bottom of a 48 well cell culture plate on the drop (20 µL) of FBS for 30 min at 30–32 °C in order to stick the sample to the surface of the well. Thereafter, 1 mL of DMEM (containing 10% FBS and antibiotics) was given to the wells (containing sterilized sample sections as well as empty wells that serve as controls) and then placed in the humidified incubator at 37 °C under 5% CO_2_ in air for 3, 5 and 7 days. The cell culture medium (sample extracts and extract control medium) was collected and used in indirect experiments.

For cell viability/proliferation study, the cells (i.e., Lep-3 and BEC) were seeded in 96-well flat-bottomed microplates at a concentration of 1 × 10^4^ cells/well in fresh DMEM medium with 10% FBS. At the 24th h, the culture medium from each well was removed and changed with 100 µL DMEM containing hydrogel scaffolds extract (sample extracts, obtained after 3-, 5- and 7-day incubation periods). The percent of viable cells was determined using MTT (3-(4, 5-Dimethylthiazol-2-yl)-2,5-diphenyltetrazolium bromide) test.

MTT test was performed as it was earlier described [33]. Briefly, the cells were incubated for 3 h with MTT solution (0.3 mg MTT in 10 mL DMEM) at 37 °C under 5% CO_2_ condition. The formed blue MTT formazan was extracted with a mixture of absolute ethanol and DMSO (1:1, *v*/*v*). The quantitative analysis was performed by absorbance measurements in an automated microplate reader (Tecan, Sunrise™, Grödig, Austria) at 540/620 nm.

#### 4.6.3. Direct Experiments (DE)

The cells (5 × 10^4^ cells/well) were seeded directly on the material sample placed on the bottom of a 48-well cell culture plate and incubated for 3, 5 and 7 days in CO_2_ incubator at 37 °C. The number and viability of the cells were determined before seeding using automated cell counter by trypan blue dye exclusion technique (zero time). The cell viability was found to be >95% in all experiments performed. At the start of the experiment the cell numbers are equal in all wells / between all different samples, and they are cultured in equal conditions. Cells were grown in wells without materials, served as controls. The effect of the materials on cell viability and proliferation was studied by MTT test as described in Section 4.6.2 with MTT concentration is corresponding to the volume of the plate.

### 4.7. Study of DNA Damages

The presence of single and double stranded DNA damages was assessed by single cell gel electrophoresis (Comet assay) at alkaline pH. Lep-3 cells were seeded in 6-well flat-bottomed microplates at a concentration of 3 × 10^5^ cells/well. At the 24th hour the culture medium was removed and changed with sample extract media (3-days modified media) and control media prepared as described in Section 4.6.2.

The Lep-3 cells were mixed with 1.4% of low-gelling agarose (Sigma Type II) and immediately spread onto microscopic slides precoated with 0.5% normal agarose. Cells were lysed for 1 h in a lysis solution (1 M NaCl, 50 mM EDTA pH 8, 30 mM NaOH, 0.1% *N*-lauroylsarcozine; pH 10). After 1-h incubation in the denaturing solution (30 mM NaOH, 10 mM EDTA; pH 12.6) for DNA unwinding, the slides were electrophoresed for 20 min at 0.46 V/cm in the same denaturing buffer. At the end of the electrophoresis the slides were subsequently dehydrated for 5 min in 75% and in 96% of ethanol. Comets were observed under Leitz epifluorescent microscope (Orthoplan, VARIO ORTHOMAT 2) using a 450–490 nm band-pass filter following staining of microgels with the fluorescent dye SYBR green I (Molecular Probes, Eugene, OR, USA). 1000 randomly chosen objects per each probe and treatment were taken for quantification. Two repetitions of the experiment were done and standard deviations were quantified. In all cases they were very small.

### 4.8. Apoptosis/Necrosis Study via Annexin V-FITC

The ability of the materials to induce cells death was evaluated by APOPTOSIS detection kit, (ANNEXINV—GFP-Certified Apoptosis/Necrosis detection kit, Enzo Life Sciences). Cells were spinned down at 400 g for 5 min at room temperature and carefully re-suspended in 1 mL cold 1× PBS (2.68 mM KCl, 1.47 mM KH_2_PO_4_, 1.37 mM NaCl, 8 mM Na_2_HPO_4_), pH 7. Spinning down follows at the same conditions and the pellet was re-suspended in 510 μL Dual Detection Reagent (500 μL 1× binding buffer, 5 μL Apoptosis Detection reagent/Annexin V-Enzo Gold; 5 μL Necrosis Detection Reagent). Samples were incubated at room temperature for 10 min at dark and were analysed via cytometry using 488 nm laser at FL 2 and FL 3 channels for apoptosis and necrosis detection respectively. Results were quantified with FlowJo software. Two repetitions of the experiment were done. Data quantitation included estimation of the percentage of cells undergoing apoptosis and necrosis and the ratio between these percentages in comparison to the control cells is given in PDU (procedure data units).

### 4.9. SEM Analysis

The samples prepared as described in Section 4.6.1. Lep-3 cells (7 × 10^5^ cells/well) were seeded directly on sample materials in a 48-well cell culture plate and left in an incubator (Thermo Scientific) at 37 ℃, 5% CO_2_. After 7 days’ incubation period, the culture medium was removed and the sample sections were washed with 4% glutaraldehyde for 1 h followed by washing with double distilled water. The samples were then subjected to dry by filter system CORNING 431097 (0.22 µm) under low pressure for 2 h and were left for 2 days at 30–32 °C for complete drying. Finally, samples were prepared for SEM analysis by a standard procedure and observed under scanning electron microscope (JEOL JSM-5510, Tokyo, Japan) at an accelerating voltage of 10 kV.

### 4.10. Statistical Analysis

The data are presented as mean ± standard error of the mean. Statistical differences between control and treated groups were assessed using one-way analysis of variance (ANOVA) and student’s *t*-test followed by suitable post-hoc test by using GraphPad Prism version 5.00 (San Diego, CA, USA) for Windows and MS Office 2010 (Redmond, WA, USA).

## 5. Conclusions

The present work focuses the biocompatibility and biological efficiency of inorganic calcium filled hydrogel scaffold in bone regeneration/bone bioengineering. The comprehensive comparative study with two cell lines indicates the cell biological efficiency of the scaffolds. Lep-3 cell line represents non-specified human fibroblast and mouse bone explant cell (BEC) line represents specified bone cells. The comparative cell viability study (with the extracts of all the six BC based hydrogel scaffolds) and biocompatibility study with two different cell lines indicates that the PVP based hydrogel scaffold can facilitate the growth and proliferation of both types of cell lines. Additionally, the percentage of DNA strand break were found less in PVP based samples than CMC based samples. Furthermore, apoptosis events were not so significantly found in Lep-3 cells with BC-PVP-β-TCP/HA. Finally, SEM study indicates the efficient adhesion and growth of fibroblast (Lep-3) cells throughout the BC-PVP-β-TCP/HA hydrogel scaffold surface. Thus, this scaffold exhibits promising osteo-conduction. Additionally, the notable cyto-compatibility of this scaffold suggests its putative ability to facilitate the process of bone regeneration. However, further studies are required with different osteogenic cell lines (i.e., osteoblast) and mesenchymal stem cell line to ascertain the efficiency of abovementioned hydrogel scaffold in bone tissue engineering.

## Figures and Tables

**Table 1:** Composition of inorganic calcium phosphate filled BC based hydrogel scaffold [48,49].

Sample Index	PVP (g)	CMC (g)	BC (g)	PEG (g)	Agar (g)	Glycerin (mL)	β-TCP/HA (g)	Water (mL)
BC-PVP	0.5	0.0	0.5	1	2	1	0.0/0.0	95
BC-CMC	0.0	0.5	0.5	1	2	1	0.0/0.0	95
BC-PVP-β-TCP/HA	0.5	0.0	0.5	1	2	1	0.2/0.8	94
BC-CMC-β-TCP/HA	0.0	0.5	0.5	1	2	1	0.2/0.8	94
